# Simultaneous Involvement of Lung and Bone Tissues in Pediatric Anaplastic Large Cell Lymphoma ALK+: A Case Report

**DOI:** 10.1155/crh/3818807

**Published:** 2025-11-10

**Authors:** Carlos Julián Díaz-Torres, Alejandra Pando-Caciano

**Affiliations:** ^1^Pediatric Hematology Service, Instituto Nacional de Salud del Niño San Borja, Lima 15037, Peru; ^2^Research and Development Laboratories, Faculty of Science and Engineering, Universidad Peruana Cayetano Heredia, Lima 15102, Peru; ^3^Subunit of Research and Technological Innovation, Instituto Nacional de Salud del Niño San Borja, Lima 15037, Peru

**Keywords:** ALK+, anaplastic large cell lymphoma, flow cytometry, osteolytic lesions, pediatric patient, pleural effusion

## Abstract

Anaplastic large cell lymphoma (ALCL) is a subtype of non-Hodgkin lymphoma characterized by the presence of CD30+ lymphocytes. While nodal involvement is common, extranodal manifestations are less frequent, with the skin being the most commonly affected organ, followed by the lungs, bones, and liver. We present the case of a 10-year-old girl who experienced a 4-month history of intermittent fever, abdominal pain, significant weight loss, and debilitating lumbar pain that restricted her mobility. Computed tomography scans performed at a national pediatric reference center in Lima, Peru, revealed osteolytic lesions primarily affecting the D12 vertebra. During hospitalization, the patient developed dyspnea and chest pain due to bilateral pleural effusions. The suspected diagnosis of ALK + ALCL was confirmed through lymph node biopsy, alongside the identification of malignant CD30+ cells in pleural fluid via flow cytometry. Following the initiation of chemotherapy, the patient experienced a complete resolution of symptoms. This case highlights the atypical simultaneous extranodal involvement of both bone and lung in pediatric ALK + ALCL, a manifestation rarely documented in the existing literature. Furthermore, it demonstrates the potential value of pleural fluid flow cytometry as a complementary diagnostic approach in ALCL, particularly when tissue biopsy is limited or not feasible. The insights provided in this report aim to assist healthcare professionals in diagnosing and managing similar cases encountered in clinical practice.

## 1. Introduction

Anaplastic large cell lymphoma (ALCL) comprises 10%–15% of pediatric non-Hodgkin's lymphomas. The presence of the CD30 antigen serves as a distinctive marker for ALCL cells and represents a relevant therapeutic target [1]. Furthermore, over 90% of ALCL cases in this age group exhibit aberrant anaplastic lymphoma kinase (ALK) activity [1, 2]. ALCL usually presents in advanced stages (III-IV) with B symptoms and mainly with lymph node involvement (> 90%) [1]. Extranodal involvement is relatively rare, primarily affecting the skin and soft tissues. Involvement of the liver (8%), lung (10%), and bone (17%) is less common [1].

Pleural effusion is a well-documented complication associated with lymphomas, and its occurrence is influenced by the specific histological subtype. Following the collection of pathologic pleural fluid via thoracentesis, a comprehensive series of evaluations, including cytological, immunocytochemical, and flow cytometric analyses are conducted to confirm the initial diagnosis derived from lymph node biopsy [3]. Bone involvement represents another rare manifestation of ALCL. In pediatric patients, the detection of osteolytic lesions through computed tomography (CT) scans may indicate the presence of a primary malignant bone neoplasm; however, these lesions can also suggest a lymphoma diagnosis [4].

We present the case of a 10-year-old girl diagnosed with ALK + ALCL, characterized by atypical involvement of both bone and lung tissues, at a national pediatric reference center in Lima, Peru. Written informed consent for the publication of this case was obtained from the child's legal guardian.

## 2. Case Presentation

A 10-year-old female patient with an unremarkable medical and surgical history was admitted to a general hospital in Lima, Peru, due to the presentation of intermittent fever, nonspecific abdominal pain, and progressive weight loss over the previous four months. During her hospitalization, analysis of cervical and inguinal lymph node biopsies were performed, revealing follicular hyperplasia without evidence of malignant neoplasia.

Due to the persistence of symptoms, the patient sought specialized medical attention at the Instituto Nacional de Salud del Niño San Borja (INSN-SB), a leading pediatric reference center in Lima. At the time of admission, the detailed reports from the referring hospital, including the specific tests performed and their results, were unavailable, and the attending physicians were aware only of the reported diagnosis of follicular hyperplasia.

To determine the origin of the fever episodes, serological tests for bacterial and viral infections were conducted, all yielding negative results. One month after these evaluations, the patient developed lumbar pain and functional impairment, prompting her return to INSN-SB for further medical assessment, where she was subsequently hospitalized.

**Figure 1:**
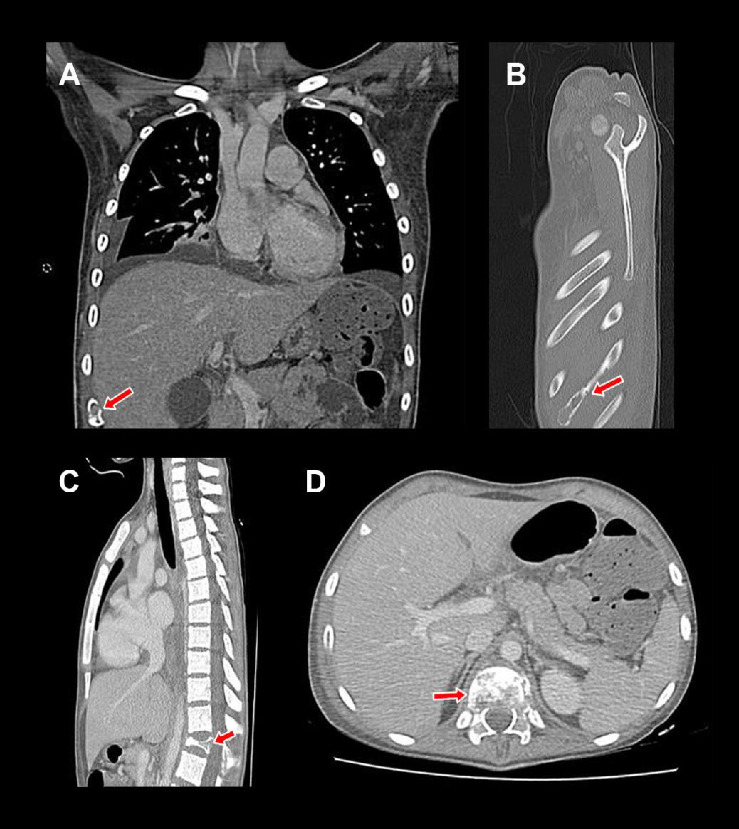
Findings from the CT scan performed upon admission to the emergency department. Lesions identified by the test are denoted by red arrows. The CT scan images revealed osteolytic lesions in the right ninth costal arch, as demonstrated in panels: (A) coronal section and (B) longitudinal section. Additionally, similar lesions were found at the level of the D12 vertebral body, resulting in compression and subsequent collapse of the vertebral body, as shown in panels: (C) longitudinal section and (D) axial section.

During the physical examination in the emergency department, nontender cervical and inguinal lymphadenopathies, measuring approximately 1–2 cm in diameter, were noted. Imaging studies did not demonstrate any evidence of hepatosplenomegaly. The motor evaluation revealed lumbar pain that impeded ambulation. Initial laboratory results showed a hemoglobin level of 8.2 g/dL, a white blood cell count of 14,690 × 10^3^/μL, an absolute neutrophil count of 12,190 × 10^3^/μL, and a platelet count of 595 × 10^3^/μL. Electrolyte levels, urea, creatinine, transaminases, and bilirubin were within normal limits. A contrasted total body tomography was performed (Figure 1), revealing the presence of osteolytic lesions in the ribs, ilium, and predominantly in the D12 vertebra.

During hospitalization, the patient was administered intravenous meropenem at a dose of 1.5 g every 8 h for 15 days, alongside vancomycin at a dose of 600 mg every 6 h for 7 days, due to persistent fever and elevated C-reactive protein levels. Sputum analyses, including tests for Koch's bacillus and GenXpert, were conducted to exclude tuberculosis, while antinuclear antibody assays were performed to assess for potential autoimmune disease, all yielding negative results. Additionally, no bacterial pathogens were isolated from the blood cultures obtained.

**Figure 2:**
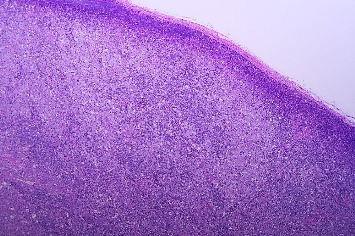
Histological section of lymph node showing disrupted architecture by infiltration of neoplastic cells (H&E stain, 4 ×).

**Figure 3:**
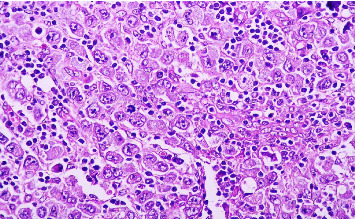
Histological section at higher magnification showing large, irregular, pleomorphic neoplastic cells with abundant cytoplasm and multiple nucleoli (H&E stain, 40 ×).

**Figure 4:**
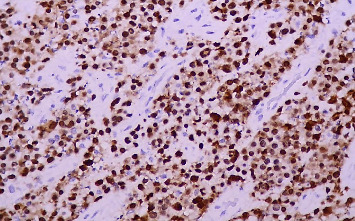
Immunohistochemical staining for ALK showing positive nuclear expression in neoplastic cells (40 ×).

Due to persistent fever and elevated levels of acute-phase reactants, a second biopsy of the inguinal lymph nodes was performed, confirming the diagnosis of ALCL (Figures 2 and 3). Immunohistochemical analysis revealed the presence of the ALK protein, characterizing the ALCL as ALK+ (Figure 4). In addition, CD30 and EMA markers were positive, while CD3 was negative. The pathology report did not specify the histological subtype or the lymph node infiltration pattern, as these evaluations are not routinely performed at the institution due to the lack of pathologists with sufficient expertise in assessing these features.

**Figure 5:**
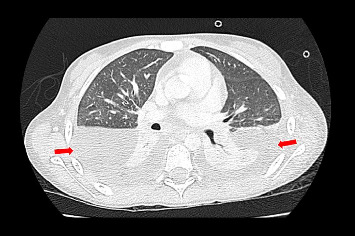
Findings from the CT scan performed during ICU stay. Extensive peripheral hypodensity was observed in the pulmonary parenchyma of both hemithoraces, corresponding with the bilateral pleural effusion indicated by the red arrows.

Following this diagnosis, the patient was scheduled for bone marrow and cerebrospinal fluid (CSF) studies to assess the disease stage. Bone marrow analysis revealed infiltration by ALCL cells, whereas the CSF showed no evidence of neoplastic involvement. However, the patient subsequently experienced respiratory deterioration, resulting in an increased requirement for oxygen over the following days, requiring transfer to the intensive care unit (ICU). Upon admission to the ICU, a chest CT scan was conducted, which revealed bilateral pleural effusion (Figure 5).

**Figure 6:**
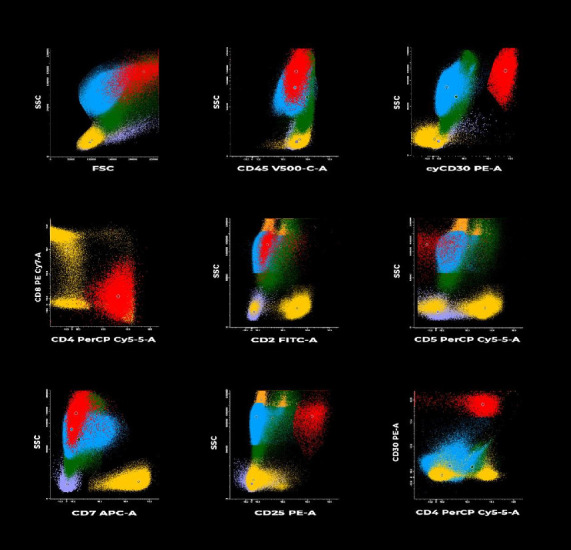
Results of flow cytometry analysis conducted on pleural fluid. Flow cytometry demonstrated the presence of 0.76% of a pathological population (indicated in red), characterized by the expression of CD45, CD30, cyCD30, CD4, CD25, and HLA-DR. No pathological cells exhibiting the markers CD3, CD8, CD7, CD19, CD33, or CD16 were detected. Light scattering parameters (FSC/SCC) revealed that the pathological cells displayed a larger size and intermediate internal complexity. A total of 2,000,000 viable events were analyzed.

The patient initiated the prephase of the ALCL99 protocol during her stay in the ICU [5]. Following treatment initiation, a thoracentesis was performed, resulting in the drainage of 350 mL of cloudy serohematic fluid. This procedure was associated with a notable improvement in the patient's clinical condition. Subsequently, the initial bone marrow assessment was complemented with a biopsy and flow cytometric analysis of the pleural fluid, which revealed infiltration by CD30+ neoplastic cells consistent with ALCL (Figure 6). Concurrently, the CSF was analyzed through flow cytometry, showing no evidence of abnormal findings.

During her stay in the ICU, the patient completed the prephase of the ALCL99 protocol, demonstrating significant improvement in respiratory function and a reduction in pleural effusion, as confirmed by chest X-ray. Subsequently, she was discharged from the ICU and transferred to the Hematology Service's inpatient unit to continue treatment, where the AM1 cycle was initiated. Following the initiation of this cycle, a marked resolution of fever, a progressive decline in acute-phase reactants, a reduction in bone pain, and an improvement in ambulation were observed. After recovery following the AM1 cycle, the patient was discharged with plans for readmission for the subsequent cycle (BM1).

Following the completion of the third cycle of treatment (AM2), the patient exhibited an increase in soft tissue volume in the right cervical region. A biopsy of this area confirmed the presence of neoplastic cells consistent with ALK + ALCL. This finding indicated a relapse of the disease, leading to hospitalization and the initiation of a more intensive chemotherapy regimen based on the NHL-BFM-95 protocol [6]. After completing the CC, AA, BB, and CC cycles, the patient achieved complete remission, with the resolution of all tumor lesions confirmed by PET scan results.

An autologous bone marrow transplant was performed to consolidate the favorable therapeutic response. No evidence of disease recurrence was observed at the 1-month follow-up after transplantation.

## 3. Discussion

Although nodal involvement is more commonly observed in pediatric cases of ALCL, extranodal involvement accounts for a significant proportion of cases. Evidence from India, Italy, and England has suggested that extranodal involvement occurs in up to 40%–60% of pediatric ALCL cases [6–8]. The case presented here illustrates simultaneous extranodal involvement in both the pleural space and bone, showcasing a diagnostic approach that integrates traditional biopsy techniques with flow cytometry.

Flow cytometry analysis of pleural fluid collected during chemotherapy revealed the presence of several surface markers, including CD45, CD30, cyCD30, CD4, CD25, and HLA-DR. Juco et al. reported that these markers, along with at least one T-cell-associated antigen (specifically CD4), were detected by flow cytometry in 100% of patients under 20 years of age diagnosed with ALCL [9]. Furthermore, CD25 and ALK markers were observed in 88% and 33% of cases, respectively. In another study encompassing pediatric and adult populations with ALCL, CD45 and CD30 markers were detected by flow cytometry in 92% of cases, while the ALK protein was identified through immunohistochemistry in 84% of individuals [10]. In the present case, the institution lacked access to flow cytometry or cytogenetic assays (such as fluorescence in situ hybridization) in tissue samples. Therefore, the diagnosis of ALK + ALCL was established based on immunohistochemical detection of the chimeric ALK protein, which reflects the NPM-ALK fusion product resulting from the t(2; 5) translocation.

The detection of tumor cells in body fluids, such as pleural fluid, is infrequently reported in pediatric ALCL. In 2020, Stephen et al. described a case involving a 3-year-old patient who exhibited lymphomatous cells in both ascitic and pleural fluid, as assessed through cytological examination. This initial finding suggested a potential diagnosis of ALCL. Subsequent immunohistochemical analysis confirmed the presence of CD30 and ALK markers, thereby establishing a definitive diagnosis [11].

Similarly, the involvement of the skeletal system in ALCL was documented by Abe et al. in a 15-year-old patient who presented with intracranial invasion [12]. Immunohistochemical analysis of the biopsy obtained from the prosencephalon demonstrated the presence of large tumor cells positive for CD30 and ALK1. Additionally, fluorescence in situ hybridization revealed an ALK gene rearrangement in the biopsy sample. Consequently, the patient was diagnosed with ALK + ALCL and successfully managed with chemotherapy based on the ALCL99 protocol.

As in the case documented here, the involvement of the D2 vertebra in ALCL has been previously reported in the literature [13]. El Farissi et al. presented a case of a 16-year-old adolescent whose spinal MRI revealed a lesion involving the D1, D2, and D3 vertebrae, with infiltration into the paravertebral muscle, as well as the spinous and transverse processes of the D2 vertebra. A biopsy of the anterior portion of the lesion was performed for histopathological evaluation, which unveiled large anaplastic lymphoid cells positive for ALK, CD30, CD5, and Ki67 markers.

An additional pediatric case of ALCL with extranodal involvement was reported by Mira-Perceval Juan et al. [14]. This case involved a 21-month-old patient exhibiting infiltration of CD30+ anaplastic lymphomatous cells in the pericardial fluid. The identification of both CD30+ and ALK + cells confirmed the diagnosis of ALK + ALCL with pericardial involvement.

Reports of pediatric ALCL in Latin America are scarce. In Mexico, ALCL was identified in over 50% of pediatric cases diagnosed with large cell lymphomas exhibiting anaplastic morphology, all of which tested positive for CD30 and ALK markers. The most common sites of involvement were peripheral lymph nodes, with only one case demonstrating cutaneous infiltration [15]. Additionally, a case involving a 10-year-old patient was documented, presenting with primary ALK + ALCL of the lung, which represents an unusual variant of ALCL. The diagnosis was confirmed through immunohistochemical analysis of an incisional biopsy taken from a mass obstructing the left bronchus and situated on the carina, revealing the presence of leukocyte common antigen, as well as positivity for the CD30 marker (Ki-1), epithelial membrane marker, and ALK1 marker [16].

Although lymph node biopsy remains the gold standard for diagnosing ALCL, cytopathological analysis of the same specimen provides critical insights into the morphology and specific immunocytochemical staining patterns of lymphoma tumor cells [3, 11]. Flow cytometry offers a complementary method for immunophenotypic characterization of lymphoma cells. When applied to pathological body fluids and interpreted alongside the morphological findings of the same samples, this technique can enhance diagnostic sensitivity and specificity and, in some instances, support a definitive identification of ALCL. In the present case, flow cytometry successfully identified neoplastic cells and supported the diagnosis process; however, the lymph node biopsy ultimately provided the definitive confirmation of ALCL.

This case also underscores the importance of timely referral from general hospitals to specialized centers for patients requiring a comprehensive diagnostic evaluation and specialized management, ensuring prompt and appropriate treatment.

## 4. Conclusions

This report describes a rare case of ALCL with simultaneous extranodal involvement of both bone and lung, a finding infrequently documented in the literature. Lymph node biopsy remains the gold standard for establishing the diagnosis. Nevertheless, flow cytometry of pleural fluid, characterized by high sensitivity and specificity and a faster turnaround than immunohistochemistry, can complement the diagnostic work-up when tissue biopsy is limited or not feasible, thereby supporting timely management. Given the uncommon presentation of ALCL with concurrent bone and lung involvement, we hope that the insights provided herein will assist other healthcare professionals in the diagnosis and management of similar cases in routine clinical practice.

## Data Availability

The data that support the findings of this study are available from the corresponding author upon reasonable request.
